# Attitudes and perceptions towards public health safety measures during a global health crisis: Social and personal consequences

**DOI:** 10.1371/journal.pone.0289357

**Published:** 2023-11-27

**Authors:** Lauren D. Terzis, Leia Y. Saltzman, J. Heath Lowman, Dana A. Logan, Tonya C. Hansel

**Affiliations:** School of Social Work, Tulane University, New Orleans, Louisiana, United States of America; The University of Alabama, UNITED STATES

## Abstract

The COVID-19 pandemic that spread throughout the globe has significantly altered our social and personal relationships. During the early phase of the pandemic, pharmaceutical interventions such as vaccine research and production were still in development, with international health agencies and governments promoting public health safety measures such as limiting mobility, school and work closures, lockdowns, economic incentives, mask-wearing, social distancing, quarantine, and hygiene to reduce the spread and flatten the curve regarding transmission and hospitalization. During the early wave (May 2020 through July 2020), we utilized a qualitative longitudinal research design coupled with weekly Zoom diary entries to investigate participant (n = 14) experiences. In doing so, we captured participant attitudes towards public health safety measures, as well as perceptions of social and interpersonal relationships during the pandemic. The main themes that emerged in our findings include feelings of safety and preparedness, personal accountability and collective responsibility, and changes to social life and relationships. While individuals have learned how to live with the pandemic, and have a new sense of normalcy, lessons learned from the impact of public health measures and social relationships have applicability moving forward post-pandemic. In particular, how to best protect against the deleterious effects of isolation during a future public health crisis.

## Introduction

In less than three years, the SARS-CoV-2 virus, which came to cause the COVID-19 pandemic, has infected 613 million people and led to 6.53 million deaths since December 2019 making the COVID-19 pandemic the 6th deadliest in human history [1]). On March 11, 2020, Tedros Adhanom Ghebreyesus, the Director-General of the World Health Organization (WHO) alerted the world as he defined the effect of the SARS-CoV-2 coronavirus as a pandemic: a disease outbreak affecting multiple countries. The response to the COVID-19 pandemic began in anticipation of the start of the pandemic. By March 2020, after two months of conducting experiments and research, numerous researchers discerned the basic features of the virus, the impending pandemic, probable routes to vaccine or inoculation development, and identified many of the preventative measures which would be prescribed for the world writ large [2, 3]. The COVID-19 pandemic represents the first time the scientific and academic community identified the pandemic before or as it was occurring, and the first coronavirus to become a pandemic [1, 4].

Pandemics have consistently presented challenges beyond viruses or bacterium to include how and what information gets disseminated, and the effect of the information on attitudes and responses to the news of the pandemic. Talking heads took to using the same evidence to propagate disparate ideologies about the COVID-19 pandemic even before the first lockdowns and recommendations from the WHO [5–9].

### Nonpharmaceutical Interventions (NPIs)

Many of the earliest safety measures were identified millennia before the COVID-19 pandemic. In reviewing historical accounts of pandemics, Vitiello and colleagues [10] propose that basic preventative measures against COVID-19 such as quarantine, social distancing, and a strong hygiene regimen are as effective and ubiquitous now as at any point in human history. In responding to the SARS pandemic in the mid-2000s, Howard Markel and David Heymann presented for the Institute of Medicine’s Forum on Microbial Threats [11] justifying advances to the International Health Regulations to detect and report looming viral and bacterial threats and implement nonpharmaceutical interventions (NPIs) such as quarantine, hygiene, and social distancing. Advances in international agreements anticipated the COVID-19 pandemic and led to directives to implement NPIs limiting the spread of the novel coronavirus through nationally directed lockdowns and quarantines [12]. While simultaneously creating pharmaceutical interventions such as vaccine research, production, treatments, therapeutics, and the global distribution thereof, the Centers for Disease Control (CDC), the World Health Organization (WHO), independent labs, giant technology firms, national and international health agencies, and governments promoted NPIs such as limiting mobility, school and work closures, lockdowns, economic incentives, mask-wearing, social distancing, quarantine, and hygiene [3, 6, 7, 13, 14].

In a scoping review of the literature on NPIs during the first year of the COVID-19 pandemic, Perra [13] found that understanding the implementation and public reception of NPIs are profoundly complex. During the first year of the COVID-19 pandemic, NPIs were the only defense against the coronavirus as the first vaccines were approved in January 2021. Moreover, NPIs do not directly affect the mortality rate from infection, but rather, attenuate the viral transmission which can reduce mortality overall [15]. The practice and use of NPIs are categorized into personal choices and policies or compulsory mandates [7, 9, 15–17]. Many analyses indicate that while compulsory NPI mandates may have stronger effects mitigating the number of fatalities from infections than personal choices to practice NPIs, compulsory mandates also increased negative emotions and resentment about NPIs, safety precautions, and possibly affected the number of people willing to make personal choices [8, 16, 18–22]. The intersection of public health information and psychosocial factors leads to attitudes, behaviors, and beliefs about the pandemic and the public’s adherence and buy-in for the practice of NPIs.

### Influences on attitudes towards NPIs in the first wave of COVID-19

The COVID-19 pandemic exacerbated the political and global turmoil that preceded it, especially in the United States [23–26]. The very fabric of our social relationships became entwined with the anxiety of being with other people. Over the course of three months, the entire trajectory of modern civilization shifted to accommodate the presentation of this coronavirus. While recognizing that NPIs could limit infection and potentially save lives, fear turned toward the economic consequences of NPIs. The economic impact of NPIs shook every facet of the global markets and trade [27]. Countries that implemented NPIs earlier witnessed fewer infections, fewer fatalities, and lower economic effects although few countries did so [10, 27, 28]. During the first wave of the pandemic, the ramifications of NPIs were presumed to be transformational at best or catastrophic at worse [28]. In many instances, public perceptions about lockdowns and social distancing were prevailingly accepted [29]. The political and economic effects of NPIs disproportionately harmed people of color, people living in poverty, service workers, laborers, and countries with lower GDPs [30, 31]. Economics, financial insecurity, rumors, and panic buying influenced public opinion about the pandemic and NPIs [32–34].

In the United States, news outlets presented the virus with political strife [23–26]. While many people recognized the danger of the virus, the pandemic was also internationally denounced as a hoax or conspiracy [23, 34, 35]. Attitudes toward NPIs became as polarized as debates on abortion, euthanasia, and the death penalty [23, 24]. The anxieties that people had and expressed about ideological strife before the pandemic grounded their fears about the efficacy and economics of NPIs [26]. Throughout the response to the pandemic, especially in the first wave, daily life meant conducting a critically anxious risk assessment of behaviors and preventing the spread of the virus even if it meant perpetual isolation from families, and careers, or being perceived antagonistically by others with opposing views about NPIs and the pandemic [23, 35].

### Attitudes & psychosocial effects of NPIs

Face masks may have been the most controversial NPI during the first wave of the pandemic. Even though face masks catalyzed the controversy around NPIs and their value, face masks were found to be effective at reducing the transmission of SARS-CoV-2 [16]. Even attitudes about face masks improved with perceived efficacy [36]. Fears about the environmental impact of disposable masks complicated attitudes towards face masks [37]. The use of face masks depends on belief in effectiveness, the ability to cope with stress, and the resolve to wear them [36]. While face masks and adherence to guidance about face masks caused increased stress and anxiety, most data on the psychosocial effects of NPIs rests on understanding isolation and its impact on mental health.

Since the SARS-CoV-2 virus is communicable through human interaction, many of the NPIs require some form of social isolation and the mental health effects that come with it. Isolation is known to exacerbate symptoms of poor mental health [38–40], especially among women and elderly people [41]. In the years preceding the COVID-19 pandemic, research on loneliness, isolation, and their effects on mental health were burgeoning following centuries of anecdotal evidence and widely held beliefs without scientific promulgation [41–43]. In the last decade, social isolation and its effects could be operationalized in a way they had not been previously. Isolation could be measured by pre-existing psychometric instruments with new insights, and isolation could be conceptualized through the quality, quantity, and structure of social networks and individual’s emotional and resource valuations of their social relationships [41, 43]. Before COVID-19, Abad et al. [42] found that isolation leads to increased feelings of anger and sadness and increased symptom severity and frequency in anxiety and depression among hospitalized patients. Interestingly, as patients were withheld from information about their conditions, their satisfaction with care and quality of life diminished [42].

The positive relationship between social support and recovery from trauma has been frequently cited in the literature [44]. A higher level of social support is a strong predictor of better mental health outcomes following a disaster [45]. More recently, social support has been found to act as a buffer to worries regarding exposure to COVID-19 [46], compensate for the negative impact on mental health among those with low levels of resilience [47], and increase the benefits of positive life events [48]. Extrapolating to the COVID-19 pandemic and its NPIs to include quarantine, community lockdowns, work and school closures, and the unreliability of the infodemic, isolation, and its effects reverberate from the first wave to now.

In February 2020, in the midst of the first wave of the pandemic, Brooks and colleagues [49] conducted a rapid review finding that NPIs such as, “longer quarantine duration, infection fears, frustration, boredom, inadequate supplies, inadequate information, financial loss, and stigma,” led to adverse psychological outcomes to include cognitive disorganization, anger, aggression, and post-traumatic stress. In the first systematic review of loneliness and the COVID-19 pandemic, Pai and Vella [50] affirmed the positive relationship between socially isolating NPIs and symptoms of mental illness. Fisher and colleagues [51] reported that coping strategies and bereavement behaviors were stunted by the stressors of the pandemic and NPIs. Further, Chhabra and colleagues [52] reported that the practice of socially isolating NPIs leads to increased symptoms of anxiety, depression, stress, post-traumatic stress, and aggression and that these increases could explain the expansion of instances of domestic violence. Sleep quality and duration also diminished as a result of stress or negative emotions caused by living through the social isolation brought on by NPIs [53]. The understanding of sleep hygiene affected the presence of disordered sleeping habits as well as anxiety and depression symptoms. The more confusion or incorrect understanding, the more likely to experience disordered sleep, anxiety, or depression [53]. Prolonged exposure to isolating NPIs leads to long-lasting psychological damage and increased symptoms of anxiety, depression, panic, somatization of physical and mental illnesses, increased reliance on substances, and negative effects on personalities would invariably increase over the duration of NPIs [54, 55]. There is also a potentially increased risk for psychological impairments following a SARS-CoV-2 infection as in SARS survivors, the compatibility of SARS-CoV-2 to ACE2 receptors in human brains provides a neurotropic effect which can lead to suicidal ideation, increased anger, and symptoms of post-traumatic stress [56].

### Current study

During the time of data collection (May 2020 through July 2020) public health officials strongly focused on encouraging NPIs to limit the spread of the COVID-19 virus. At this time, COVID vaccines were not developed or available, thus the reliance was on NPIs to reduce the spread and flatten the curve regarding transmission and hospitalization. The purpose of this study was to investigate participant experiences during the early wave of COVID-19, and in doing so we captured their attitudes towards NPIs and perceptions of social and interpersonal relationships that were impacted during the early wave. This paper is guided by two research questions: (1) What are the perceptions and attitudes toward NPIs during the COVID-19 pandemic? and (2) How has the pandemic and utilization of NPIs influenced social interpersonal relationships?

## Materials and methods

The current study used a qualitative longitudinal research (QLR) design, with Zoom diary entries as a way to collect data from participants. Participants were recruited via student listserv email from a graduate program (Masters of Social Work) at the Authors’ institution. Inclusion criteria for participation included 1) being 18 years or older, 2) comfortability speaking English, and 3) access to Zoom technology. Participants completed up to eight diary entries over the period of eight weeks, from May 2020 through July 2020. Guided prompts were provided to participants to engage in thought and self-reflection of the week, as well as adaptations and changes over time that they experienced due to the pandemic. Due to the use of human participants, review and approval for this study was obtained from Tulane University’s Human Research Protection Office (Tulane University IRB # 2020–505) before the study began. Participant informed consent was obtained online via a consent script through the Qualtrics platform. Participants were able to read the consent script, pause their participation and contact the research team for clarification on the study before consenting to participate. Consent was obtained by clicking on the “I consent” button at the bottom of the consent form. Participants who did not wish to consent selected “decline” and exited the survey. Once consent was obtained participants were routed to the survey. No minors were enrolled in the study, all participants were 18 years of age or older.

### Measurement

Participants completed a one-time online survey including a screening for anxiety (GAD-7; [57]), food access [58], use of substances (CAGE; [59]), social interdependence [60] (Gerpott et al., 2018) and basic demographic information [61]. Participants were then invited to eight recurring ZoomTM meetings using individual links (one time per week for eight weeks consecutively). All ZoomTM sessions were recorded though participants had the choice to provide video and audio, audio only, or typed text based on their preference and comfort level [61]. Participants received the following prompts to frame their diary entry each week: (1) One a scale of 1–10 where 10 is the best you have ever been and 1 is the worst you have ever been, what number would you rate yourself today and why?; (2) Describe what life has been like this week; (3) What changes (either positive or negative) have you made since last week as a result of the COVID-19 pandemic?; (4) What about these changes has been easy to cope with?; (5) what about these changes has been difficult to cope with?; (6) What is one thing that would help you cope better? And (7) is there anything else you want us to know about life during the COVID-19 pandemic? [61]. All study activities took place online as was necessary during the early stages of the COVID-19 pandemic. The length of each diary entry varied between participants and between entry weeks, and ranged from 5 minutes to 45 minutes in length.

### Trustworthiness and rigor

Several strategies were employed by the research team to ensure trustworthiness and rigor of the study. First, observer triangulation was used, where three members of the research team met weekly to read through the transcript and code all the data. The three research members met to discuss application of codes, as well as resolution of any discrepancies among the coding. Second, as the global pandemic was currently impacting the researchers in real time, the authors engaged in reflexivity in order to reflect on their own experiences and biases regarding the pandemic. Third, the researchers kept a detailed audit trail with thick descriptions throughout the data analysis process.

## Results

A total of 14 participants recorded diary entries on Zoom throughout the data collection period (8 weeks). Thirteen of the participants identified as female, with a mean age of 27 years (*SD =* 4.68). In terms of race, 9 participants identified as white, 4 as Black or African American, and 1 as Asian. All participants were graduate students at a private university located in the southern region of the United States.

For diary entry completion, 2 participants completed the full 8 diary entries, four completed at least 6, and a total of 8 participants completed at least half (4 diary entries). This accounted for 54 diary entries to be coded and analyzed by the study team. A team of three researchers met weekly to review the Zoom diary data to check for content safety and accuracy of automatic transcription. Coding of the diary data was conducted as a group, where the three researchers met and discussed the transcript and coded line-by-line, utilizing the appropriate application of codes and sub-codes from the codebook. When a discrepancy in coding emerged, the researchers discussed the definition of the code and its applicability until a group consensus was reached. Codebook revision was an iterative process, and the codebook developed over time as more diary entries were coded. After the codebook was finalized, the second round of coding was conducted to ensure that all appropriate codes were applied to all 54 diary entries. Dedoose, a web-based qualitative analysis software program, was used to analyze the diary entry data [62].

Several themes and subthemes were constructed from the qualitative data analysis. These included: Theme one: Safety–a) preparedness, b) stressful setting, and c) concern for others/vulnerable population; Theme two: Personal accountability–a) precautions, b) responsibilities, c) judgment, and d) contagion anxiety. Theme three: Social interpersonal–a) social isolation, b) changes to social life, c) desire for connection, and d) support. Table 1 includes the study’s full codebook and provides each theme and subtheme, its definition, and an example quote.

**Table 1:** Themes, sub-themes, definitions, and examples quotations.

Theme	Sub-Theme	Definition	Example
Safety			
	Preparedness	Participant discusses their preparation and safety steps in venturing out of their home (bringing sanitizer, certain times of day, wearing mask, social distance 6 feet)	*And now I don’t because the anxiety of having to have everything perfect so that I’m safe*. *Do I have my hand sanitizer*. *Do I have my mask*, *I carry a paper towel so When I leave don’t touch anything*. *Where am I going*, *what will I be doing*. *Do I need wipes*. *Do I need You know*, *how can I be best prepared*. *You can’t prepare for something like this*, *and my type A personality wants to prepare and ensure safety*,
	Stressful Setting	Participant discusses situation or location that they find stressful or unsafe due to exposure to COVID (for example medical, grocery store internship, classes)	*I went to the store last week*, *and there were over 50 people in Walmart and I freaked out*. *I had a panic attack um my heart started racing*.
	Concern for others/vulnerable populations	Participant discusses their concerns they have for family/friends/vulnerable populations and the impact of COVID on them	*I didn’t get to go see my grandma*. *Supposed to do that and April*, *and she is frail*, *so I didn’t want to risk anything*, *I could never forgive myself for that*.
**Personal Accountability**			
	Precautions	Participant discusses responsibilities one most undertake to limit the spread, and their reactions when people do not.	*I just continue to wear a mask and I continue to wash my hands daily and to make sure that I just follow precaution with everything*.
	Responsibilities	Participant discusses a collective responsibility to one other (individualism/community approach “all in it together”)—we all have a responsibility	*other people not taking it seriously and me feeling like I’m out of control because I don’t have an ability to control other people*. *I’m not responsible for them*. *But you know*, *it’s going to take all of us to beat this*
	• Judgment ^a^	Judging others not following procedures and protocols, wearing masks	*I went to the beach*. *I saw a group of people*. *I stayed away but I saw people playing frisbee and I was just really disappointed*. *I just don’t think People are being as careful as they need to be because we haven’t been through something like this before*.
	Contagion Anxiety	Participant discusses fear of spreading to others without knowing.	*And I’m scared of passing it to other people and not knowing about it*.
**Social Interpersonal**			
	Social Isolation	Participant discusses the logistics of being in isolation	*Changes that have been difficult to deal with is working from home and being more socially isolated*.
	• Family ^a^	Participant discusses being isolated and/or far from their family	*COVID has exasperated my loneliness down here*. *I live essentially by myself as I mentioned before*, *Way too far away from my family for an international disaster*.
	Changes to social life	Participant discusses what is missing in their social life? What is gained?	*It’s really hard not to have that same like ability to hug your friends and see your friends without the internal stress of the pandemic*. *And so I can’t interact with them the same way that I really want to*
	• Physical Touch ^a^	Participant discusses physical touch	*not being able to hug my grandma*
	Desire for connection	Participant discusses the desire for community and connection. Wants to feel connected, or how good it feels to be connected	*I’m just sitting around and because of COVID I can’t make other connections*. *I can’t go out and talk to other people*. *And make new friends*. *And that’s just really bugging me because I want to make new connections and meet other people*.
	Support	Participant discusses support, and feeling supported by others (such as professors, family etc). Also could refer to the desires and/or changes in support that one is experiencing.	*I Just take it day by day and I Like I talk about it with friends and family members and like it’s helping me when we talk about it in classes*

^a^ The sub-codes Family and Physical Touch appear throughout participants’ responses with sufficient data to mark a distinction but insufficient data to denote a new code.

### Theme one: Safety

While participants were not directly asked to reflect on their practices and feelings regarding safety during the pandemic, topics regarding safety organically arose in the individual diary sessions. Subthemes of safety included: 1) preparedness, 2) stressful settings, and 3) concern for others (particularly vulnerable populations such as the elderly and immunocompromised individuals).

#### Preparedness

In the diary entries, several participants discussed ways in which they prepared themselves and the steps that they took to ensure safety during the early phases of the pandemic. Many participants discussed the NPIs that they utilized when they had to venture out such as hand sanitizer, face masks, gloves, and disinfectant wipes. As well as the other steps to ensure their safety, which included, adhering to the advice from public health authorities, engaging in social distancing (at least 6 feet), home isolation, avoiding individuals not wearing face masks, and only venturing out at certain times of the day. One of the participants discussed utilizing a checklist of items to ensure their safety and so that they were prepared when leaving their home: “Do I have my hand sanitizer? Do I have my mask? I carry a paper towel so when I leave I don’t touch anything” (participant 7). While some participants discussed adhering to stay-at-home orders and working/studying from home, others discussed ways they kept themselves prepared and safe at work. One participant discussed their experience and how their preparedness interacted with relationships at the workplace: “at my office if somebody doesn’t have on a mask, they cannot come in my office. I just can’t, I don’t want to” (participant 2).

#### Stressful setting

Throughout the diary entries, participants regularly identified situations and locations that made them feel unsafe and invoked feelings of stress. Locations that were anxiety-producing and stressful for participants that were mentioned included: the grocery store, going to a place of work or school/internship, the hospital, and another person’s home. The main stressor of these locations was the close proximity that people were to one another, as well as the concern to be around others that were not wearing masks or practicing social distancing. One of the participants shared how stressful locations and settings have impacted their social life:

I used to be a social outgoing person and I would go everywhere out to eat you know out to lunch with friends… I’m not doing any of that now because [there are] too many people. I went to the store last week, and there were over 50 people in Walmart and I freaked out. I had a panic attack, and my heart started racing (participant 2)

Other stressful activities included traveling (such as road trips and via airplane), leaving the house and being in public, being within six feet of another person, and having people that did not reside in the household visit. Many of the participants identified that a source of their anxiety in these stressful settings was that others were not utilizing NPIs: “nobody’s wearing a mask and it’s given me a lot of anxiety” (participant 9).

#### Concern for others and vulnerable populations

The majority of participants indicated that they felt a major concern for others during this time, such as vulnerable, elderly, and immunocompromised family members, friends, and community members. In particular, several participants discussed their concern for older adults in their family, such as their grandparents. One participant shared their concern and worry: “I didn’t get to go see my grandma. [I was] supposed to do that in April, and she is frail, so I didn’t want to risk anything. I could never forgive myself for that” (participant 7). One of the main concerns associated was unknowingly passing along the virus and being “asymptomatic”. Another participant shared their concern about the implication of asymptomatic spread in the community: “Because people who are asymptomatic may be passing it on to people who are at high risk and those high-risk people may not have the resources to get medical care” (participant 9).

### Theme two: Personal accountability

Similarly related to safety concerns was one’s own personal accountability during the pandemic. Several sub-themes emerged including a) precautions, b) responsibilities, c) judgment, and d) contagion anxiety.

#### Precaution

All of our participants discussed the ways in which they undertook personal precautions in how they limited the spread of the virus. Similar to safety measures, the majority of participants indicated NPI use, such as wearing a face mask in public, following social distancing guidelines, not going out in public, not allowing visitors to their home, temperature checking, limiting visits with older adults and immunocompromised family and friends, eating out less, and reducing travel. One participant discussed the precautions that they took when engaging in what they considered a “safe” social interaction with a friend, and how this social interaction benefited their feelings of social isolation:

[One change] I made since last week is I hung out with a friend. We were like, careful and we took as many precautions as we could, but it was nice to not just be by myself and to actually see somebody else and talk to somebody else in person (participant 12)

#### Responsibilities

Participants also discussed the collective responsibility people have, as a community, to overcome the pandemic together. This sub-theme emerged from participants’ sense that it takes a community response, and an “all in it together” approach to reduce and limit the spread of the virus. Many of the participants acknowledged that everyone needs to make a concerted effort in working together and to do their part.

That’s been a big theme of COVID is like I’ve said, like other people not taking it seriously, and me feeling like I’m out of control because I don’t have an ability to control other people. I’m not responsible for them. But you know, it’s going to take all of us to beat this and my look out on it is if you’re not on the team then like, get off like, we’re all on a team here. And if you’re actively trying to work against or you’re on the other team, like you can walk, like I don’t care. But you’re going to get hurt in this, and I don’t want anyone to get sick, but you’re making it worse (participant 7)

Among participants, there were feelings of resentment and frustration towards others that were not taking their responsibility of reducing the spread seriously. Another participant shared their experience and feelings regarding concern for vulnerable individuals: “I work with an elderly population and people with compromised health conditions. And so when I see people not wearing masks, not social distancing, it’s very disturbing to me” (participant 4).

#### Judgment

The frustration of others not engaging in the need for collective action to overcome the pandemic led to several participants discussing their judgment of others. This included judging those individuals that did not follow protocols and precautions to limit the spread of the virus, such as wearing face masks, social distancing, or following stay-at-home guidelines. A participant stated they found it “really difficult to be kind towards others that are not following the same social distancing protocols” (participant 4). Another participant discussed how they had family members who were not following protocol, and this made them cautious of seeing them, and that it impacted their own mental health. Feelings of frustration towards a family member appeared to strain the relationship, especially due to the differences in attitudes towards safety personal accountability.

My brother is coming into town this weekend, but he’s from Denver, so like I want to see him because I don’t see him ever, and frustrated because he’s coming home, and he’s going to have friends over, and I think that’s really inappropriate considering COVID. Which irritates me, and I think it speaks a lot to the lack of control how other people are treating this pandemic, that really irks me and really frustrates my mental health (participant 7)

#### Contagion anxiety

There was also a sense of anxiety among participants in the study, about the fear that they had about spreading the COVID-19 virus to others, and specifically spreading it to others without knowing if they were infected. Many participants described the “stress” that they incurred and the worry that they were contagious and “could be carrying the virus to others” (participant 13). During the time of data collection, media and news outlets had indicated that it was possible that individuals could be infected with the virus, yet not have any symptoms. Participants described “worrying about others” and the guilt that they would feel, particularly if they were in fact asymptomatic and contagious, and what would happen if they unknowingly passed it on to a family member or friend that was elderly or immunocompromised. These thoughts impacted relationships with family members and friends as there was a need to “protect them at all costs” (participant 2). Many of the participants discussed how the anxiety of spreading the virus to others limited their social interaction with family and friends, and instead of risking meeting in person, participants discussed ways that they connected virtually instead, to avoid potential transmission.

### Theme three: Social and interpersonal

Throughout the course of the diary entries, it was evident that participants’ social and interpersonal life was heavily impacted by the pandemic and the utilization of NPIs. Subthemes that were constructed within the social interpersonal theme include: a) social isolation, b) changes to social life, c) desire for connection, and d) support.

#### Social isolation

The majority of participants indicated that social isolation was experienced throughout the two-month period of data collection. Social isolation stemming from the COVID-19 pandemic appeared across all participants, as well as across time. Many participants discussed feeling “alone” and “socially isolated” due to the restrictions, such as stay-at-home orders that were in place to limit the spread. In particular, participants discussed being physically isolated (in their place of residence), and also in the context of being isolated from family and friends. Social distancing orders included workplace and educational closures, and participants were experiencing feelings of isolation related to working from home and attending school virtually. One participant discussed how socially isolating during this time impacted the new relationships and connections that they hoped to make after moving to a new city for a university graduate program.

It’s been hard to cope with that because I’m just sitting around and because of COVID I can’t make other connections. I can’t go out and talk to other people and make new friends. And that’s just really bugging me because I want to make new connections and meet other people (participant 9)

Some participants indicated that they were single and identified that being alone during the lockdown phase of COVID was particularly challenging, with their only connection being via technology. For example, a participant shared that “I’m single, I’m out here by myself, and I don’t have a support network at all. All my support network is the screen or through a phone call, and that’s a shitty feeling” (participant 7). Most participants spoke about how they missed their *family* during the pandemic, and that being away both geographically and physically was “stressful” on their mental health and coping.

#### Changes to social life

Within the social interpersonal theme, many participants discussed the challenges and changes that they experienced in their social life during the first wave. The most common change discussed was not being able to physically see family and friends, as well as missing out on *physical touch* with others.

I saw my grandparents for the first time because they live in New Orleans, and it was so hard, and having to sit on the porch like 20 feet away from each other. And it was such a gift to get to even talk to them in person, but it’s really hard when [you] can’t hug your grandma and she’s over there saying ‘I just want to hug and kiss you right now’ (participant 4)

Despite the changes and lack of physical interaction with family and friends, participants also discussed how they were able to safely connect during this time. This included engaging in social distancing activities, gathering outside, and connecting via technology. Many of the participants discussed how they used video conferencing technologies such as Zoom and Skype, as well as using video calling over the cell phone (FaceTime, WhatsApp, etc.) to connect with family and friends.

#### Desire for connection

The desire for connection and community emerged as a need for many of the participants. The difficulties experienced with lack of connection, specifically the need for “socialization”, “social engagements”, and “outlets”, as well as the ability to “go places” were what was missed among participants. One participant discussed the challenges that social distancing and the pandemic had on relationships with others: “It’s so hard to act in a COVID context, just with controlling crowds, but also just like your loved ones. I have friends that I can’t console, that I can’t go just like take their mind off things as we can’t gather together” (participant 7). Further, several participants also discussed their belief that being able to socialize with others would help them to “cope” that week. Once participants were able to safely connect, whether physically or virtually, many of them described the positives and enjoyment they experienced. For example, a participant discussed their experience after finally seeing a friend in person: “it was nice to not just be by myself and to actually see somebody else and talk to somebody else in person” (participant 12).

#### Support

Many of the participants also discussed the ways in which they felt supported, through family, friends, co-workers, and their professors, as well as the times that they did not feel supported. In terms of their ability to cope during the pandemic, several participants noted that feelings of support were very much needed during this difficult time: “I need support, and I’m not getting it from the city. Support [would be] what helped me cope better” (participant 7). Further, support from friends, even those that are afar has been helpful: “a lot of communication with my family and friends back in Texas, which has been awesome” (participant 4).

## Discussion

This study aimed to investigate experiences during the early wave of COVID-19 (May 2020 through July 2020), and in doing so, we were able to capture participant attitudes towards NPIs and perceptions of social and interpersonal relationships during this time frame. Since our diary study collected data from an early period of the pandemic, discussions of vaccines to curb the spread of COVID-19 were limited, and only in the context of participants reflecting on the possibility that a vaccine could be developed in the future. Thus, the focus of reflection among our participants was on early safety measures and NPIs that were mandated to limit the spread of the virus. All of the participants in our diary study stressed the importance of public health measures, such as the utilization of NPIs to ensure the *safety* of both themselves and others during the pandemic. Our participants discussed NPIs that they utilized and that were promoted by the CDC, the WHO, as well as other pharmaceutical companies, and public health authorities. NPIs discussed in the diary entries reflect previous literature and included quarantining, hygiene practices, social distancing, wearing face masks, lockdowns, as well as school and work closures [3, 6, 7, 13, 14]. Generally, our participants had a positive attitude towards the policies that were in place (wearing face masks, moving from in-person to online work/school, lockdowns, etc.) as they saw these measures as a way to keep the community safe. In particular, we were able to see changes over the 8 weeks of data collection, as some participants were able to accept their “new normal” and adjust, while some participants continued to struggle with adaptation.

Our participants also all had a sense of *personal accountability*, meaning that they were consistent users of NPIs, and adhered to government policies and mandates, such as engaging in home quarantine and isolating if need be. None of the participants stated their opposition to the policies in place (such as mandated mask-wearing in public, at indoor venues, and during transit–such as airplanes). However, they were in regular contact with other individuals who opposed or did not follow policies, which severely impacted their relationships with those individuals. Most commonly, participants discussed family/friends/co-workers, as well as the public that did not adhere to safety practices. Attitudes towards those that did not practice safety measures and utilize NPIs were mixed, but generally, participants felt frustrated with those not following protocols. For example, several participants expressed disappointment and frustration when family and household members were not engaging in social distancing, or wearing masks out in public. In one instance, a participant had a particularly challenging time with their sibling visiting from another state and inviting their friends over, defying the social distancing protocol in place. Frustration also occurred when out in public, when seeing large groups gathering and in close proximity to one another. These instances were challenging due to the participants’ own views on *personal accountability* in limiting the spread. In many instances, the consequences included participants limiting physical contact with others that were not within their household. It is important to note that during the early stages of the pandemic, there appeared to be conflicting and confusing guidelines, as well as a proliferation of misinformation being disseminated through various forms of media. Several participants discussed the tension that existed, especially with their family and friends that had opposing views about NPIs and the pandemic. Similar to previous research, our participants discussed the tension between conflicting viewpoints, and how it impacted their personal and social relationships with those individuals, even if it meant being perceived as antagonistically and being isolated from family and friends [23, 35].

Our participants’ *social and interpersonal* relationships with others were impacted heavily throughout the data collection period. All of our participants discussed how their social life has been impacted and changed because of the pandemic. What was most frequently discussed was feelings of social isolation, and how each participant managed and coped with feeling alone. To combat loneliness and feelings of isolation, our participants turned to virtual means to connect with others, such as Zoom, FaceTime, etc. Participants also discussed other ways they could interact with their loved ones in person, such as meeting outdoors and being at least six feet from one another while also wearing a face mask at all times. Many of our participants that remained connected to friends and family (virtually or from a distance) acknowledged that the social support they received significantly helped with their mental health, whereas those participants that reported a lack of support tended to discuss negative mental health experiences [45]. In our study, it was evident that receiving social support also acted as a buffer regarding concerns related to exposure to COVID-19 [46]. While most of the participants discussed social isolation and the desire to feel connected with others, we had a select few participants that expressed feeling trapped and increased tension in their place of residence, particularly if they were residing with others, such as family or roommates, placing further strain with those that they lived with. Meaning that not all participants felt the negatives of social isolation and the desire to connect with others during this time.

Similar to previous research on isolation and mental health, it was evident that our participants experienced negative psychosocial effects during this time [38–40]. Many of our participants discussed feeling stressed, anxious and depressed each week as a result of social isolation and the unknown of the COVID-19 pandemic [52]. Some participants also indicated somatic complaints, such as back and neck tension [53]. While feelings were exacerbated during this difficult time, our participants were able to discuss ways that they were able to cope with their mental health. This included self-care practices, such as processing and connecting virtually with others, picking up new hobbies (and restarting old ones), being out in nature, and having access to a therapist. One participant stated that participating in this diary study and recording weekly entries was a cathartic process that helped the process their weekly emotions.

While our data captured the trajectory of experiences in the early phases of the COVID-19 pandemic, none of us could have foreseen how COVID-19 would evolve over the next three years. Participants frequently reported “waiting” for the vaccine and for the number of infections to decrease in the hopes that a return to pre-COVID life would be possible. Since that time, we have seen several surges of COVID-19 variants with variable outcomes related to infection, the severity of illness, hospitalization, and death. Similarly, a vaccine has become available in the United States–and despite some degree of vaccine hesitancy, the majority of the US population (over 80%) has been vaccinated for COVID-19 [63]. In many ways, day-to-day life has returned to “normal” but for some individuals NPI behaviors (e.g. masking, quarantining while sick, and social distancing) remain important to their ability to comfortably interact in their workplaces, schools, and social arenas; particularly for vulnerable sectors of society (e.g. chronically ill, older adults, young children).

Furthermore, the long-term impacts of the period of time during which individuals were socially isolated continue to unfold, even for those who have returned to typical daily life [38]. Finally, a new challenge has emerged in the wake of the worries regarding acute COVID infection; long COVID syndrome. Long COVID refers to individuals who do not resolve their COVID-19 symptoms within 12 weeks of their acute infection [12]. While long COVID is not thought to be contagious, its debilitating effects on the body do require that individuals take additional precautions from re-infection and can result in stigma, isolation, and disruption to work/school, familial, and social life [64]; suggesting that understanding the attitudes towards NPI as well as the short- and long-term effects of NPI on social and personal relationship remains an important area of investigation.

### Limitations

Three limitations should be considered when interpreting these findings. First, as is the nature of qualitative research, one major limitation of the study is the small sample size limiting generalizability. The results reflect the experiences of 14 graduate students enrolled at a university located in the southern region of the United States, however, we note that while our sample was small, we coded 54 unique diary entries and reached saturation in the development of our codebook. Secondly, we acknowledge that our data was limited to the early phases of the COVID-19 pandemic and did not include additional diary or survey follow-up. While this limitation does not allow us to understand the current (almost 3 years following the start of the pandemic) experiences of participants impacted by COVID-19, it does provide insight into the confusion that dominated the early stages of the pandemic. Better understanding the failures and successes during this stage has important implications for how we address future health crises. Finally, we note the compounding nature of other world events that were taking place at the same time (e.g., the murder of George Floyd, a Black man killed by a white police officer on May 25, 2020, in Minneapolis, United States) that likely influences our participants’ need to engage with colleagues and friends for support–possibly increasing the emphasis on the social and interpersonal isolation experiences by our participants.

Despite the limitations, this study has several strengths that must be highlighted. The main strength of this study was the utilization of an innovative virtual diary data collection method that quickly and safely assessed social work students’ reactions to COVID-19 during the early phases of the pandemic. Because this study was launched during the initial phases of the pandemic, the findings reveal early coping and adaptation to abrupt change between May 2020 through July 2020. Additionally, the data reached inductive thematic saturation. Over the study period, 54 diary entries were submitted resulting in an abundance of data. This paper presents only a subset of the findings. Finally, the weekly timeframe selected likely limited retrospective effects [65].

### Implications

While individuals have learned how to live with the pandemic, and have a new sense of normalcy, lessons learned from the impact of NPIs and social relationships have applicability moving forward post-pandemic. In part, this is because the long-term impacts of the period during the COVID-19 pandemic that required near total social isolation and hypervigilance regarding NPIs to reduce the spread of COVID-19 are long-lasting, and understanding risk and protective factors that promote positive outcomes despite these extreme measures may aid in building resilience for future crises. Furthermore, while the need to utilize NPIs is no longer as pressing for the majority of the U.S. population, it still remains a critical safety measure for some vulnerable groups within society.

Moving forward, the COVID-19 pandemic and the responses by different countries and regions has demonstrated ways that leaders and policymakers can strategize to mitigate the potential of future pandemics. In particular, strategies such as policy implementation for the prevention and containment of a future pandemic are vital. With an abundance of published research on prevention and mitigation strategies now available, policymakers and leaders are well-equipped with up-to date resources and evidence to manage when a pandemic occurs again in the future. Policy implementation is not only important at the government level, but also at the institutional level and in the workplace. Our participants discussed how they were impacted by the policies from a government level and also from an institutional/workplace level. Therefore, institutional and community leaders would also need to ensure that they are prepared with appropriate policies should a pandemic occur again, in order to further protect their employees, clients, and community members that they serve. In addition, ways to address and prevent the negative effects of social isolation must also be considered, potentially through supportive programming and access to mental health services.

## Conclusion

Understanding the perceptions and attitudes toward NPIs is critical to prepare for future health crises. The polarization regarding public health practices has resulted in hesitancy, and in some cases, mistrust, of public health and medical agencies which has impacts that far outreach the COVID-19 crisis. As such, we urgently call on scholars to continue to investigate the ways in which attitudes and perceptions influence safety behaviors and how best to protect against the deleterious effects of isolation during health crises.
